# The College of Physicians

**Published:** 1833-10

**Authors:** 


### MEDICAL POLITICS AND INTELLIGENCE.

### I. THE COLLEGE OF PHYSICIANS.

**The following petition was presented to the House of Commons, towards the close of the late session.**

To the Honourable the Commons of the United Kingdom of Great Britain and Ireland, in Parliament assembled.

The Petition of the undersigned Physicians, practising in London, humbly sheweth,

That the charter of the Royal College of Physicians of London was granted by Henry VIII., for the advancement of medical science, and for the protection of the public "against the temerity of wicked men, and the practice of the ignorant."

That six physicians were named in the charter, who, together with all men of the same faculty then resident in London, were constituted one body, commonalty, or perpetual college.

That the perpetuity of the college was to be kept up by the future admission of all men of the same faculty into the college.

That several of the six physicians named in the charter studied at, and possessed degrees from, foreign universities; and that no distinction is mentioned, as regards the university where a physician may have obtained his degree.

That all physicians entitled to practise in London are equally entitled, under the charter, to admission to the fellowship of the college.

Your petitioners are prepared to show that by-laws have been framed, and long acted upon by the college, which are directly opposed to, and in violation of, the letter and meaning of the said charter.

That the physicians practising in London are invidiously divided by the by-laws of the college into two orders: one is denominated fellows; the other, constituting by far the majority, is designated (and by implication degraded) by the term licentiate.

That the fellows have usurped all the corporate powers, offices, privileges, and emoluments attached to the college; that the licentiates do not participate in these benefits, but are illegally excluded from all the offices, and any share in the management of the corporation; and so far is this principle of exclusion carried, that the licentiates are not even admitted to the library or museum of the college.

That there exists no foundation in the charter, or in the acts confirming it, for such distinction of orders, and consequent exclusion from all privileges.

That, according to one of the by-laws, no physician can claim admission as a fellow, unless he has graduated, or been admitted *ad eundem*, at the universities of Oxford or Cambridge, where medicine is imperfectly taught; while physicians who have graduated at the other British or foreign universities, celebrated as schools of medicine, are unjustly excluded from the fellowship by this obnoxious by-law.

That the college was admonished from the bench, by the Lord Chief-Justice Mansfield, to amend their by-laws in reference to the admission of licentiates into the fellowship; that, influenced by this censure, the college framed other by-laws, deceptive in their character, which, whenever they have been acted upon, have tended still further to depress and injure the order of licentiates.

That the college demand and receive a large sum of money from the fellows and licentiates, for the supposed privilege of practising as physicians within a circuit of seven miles round London, and that they do not, and cannot, protect them in this privilege.

That the graduates of Oxford and Cambridge are obliged to be members of the established church of England, and consequently all dissenters are excluded from claiming the fellowship: this your petitioners consider as a grievous injustice, and an act of intolerance unbecoming the present age.

That these invidious by-laws, made in the spirit of corporate monopoly, have involved the college in continued litigation, and created a jealousy between the fellows and licentiates, discreditable to the members of a liberal profession.

That your petitioners with deference submit, that the College of Physicians, as at present constituted, is wholly inadequate to the due regulation of the medical profession in this country, and the protection of the public; and further, that the charter of the college in no way provides for the practice of physicians in the several counties of England and Wales.

Confiding in the wisdom of Parliament, your petitioners therefore pray, that your honourable House will institute such inquiry into the state of the medical profession in this country, and the College of Physicians in particular, as will lead to the framing of laws by which the evils complained of may be removed.

And your petitioners will ever pray, &c.

Gilbert Blane,
Henry Clutterbuck,
George Birkbeck,
W. Somerville,
Alexander Morison,
Thomas Brown,
Alexander Henderson,
Charles F. Forbes,
Charles Locock,
Neil Arnott,
Roderick Macleod,
John Veitch,
W. Gairdner,
William Russell,
Hugh Ley,
James Clark,
Robert Lee,
Marshall Hall,
William Whymper,
Thomas Hodgkin,
C. J. B. Williams,
Alexander Tweedie,
Henry Davies,
J. W. Crane,
Theodore Gordon,
Whitlock Nicholl,
A. T. Thomson,
John Sims,
James Copland,
George Gregory,
J. C. Somerville,
James Bartlet,
John Webster,
Thomas Harrison Burder,
Thomas Davies,
T. Southwood Smith,
David Barry,
Charles Holland,
John Foley,
Francis Boot,
R. M. Kerison,
J. C. Roberts,
William Stroud,
James Johnson,
Edward Rigby,
Robert Richardson,
G. G. Sigmond,
James Hope,
A. T. Holroyd.

It is impossible to deny this document the praise of being exceedingly well drawn up; it is smooth in its style, plausible in its argument, and admirably calculated to make a deep impression on those members of parliament who are but imperfectly acquainted with the state of the medical profession; in plain English, on the whole house, with the exception of half-a-dozen members. There are, however, a few objections which may be made to this petition, so obvious that we should have thought it unnecessary to state them formally, had it not been taken for granted in every commentary that we have hitherto seen, that the petition was not merely an able piece of special pleading, but was perfectly unanswerable.

In the first place, if the fellows have usurped the power which they exercise, and if those oppressed ("and by implication degraded") beings, the licentiates, desire nothing but the letter of the charter, why apply to parliament? Every lawyer would tell them that the Court of King's Bench is the place to seek redress. We suspect the reason of the coy reluctance of the licentiates to pour their complaints into legal ears to be this: they would learn that the by-laws are not only consonant with the letter and spirit of the charter, but that in a modified form they are necessary to the welfare of the college. Three centuries ago any one invested with the degree of M.D. was admitted a fellow of the college, say the petitioners. Perhaps so. But was the degree of M.D., in the days of his late majesty, Henry VIII. scattered in such profusion among the population of these realms? Was the prodigious fertility of Edinburgh, the liberal facility of Aberdeen, then known? The fact is, that these and other universities have destroyed the value of any distinction founded on the degree of M.D., which is in the present age a mere play upon words; and the use of this historical legerdemain shows a lamentable weakness in any argument which depends upon it.

The Medical Gazette, and indeed the whole of the medical press, with a prudent reserve which is abundantly amusing, have entirely abstained from giving the reasons which may probably have influenced the fellows of a former day when they enacted the by-laws in question. To us it seems most likely that they were passed when the northern seminaries of physic had just discovered the advantage of diploma-making, when Aberdeen, to use Dr. Johnson's pun, was beginning to grow rich by *degrees;* and when the fellows saw that if these titular honours were allowed to pass current at their nominal value, the college would speedily be swamped with Scotchmen. We anticipate that some will admit the consequence, but will ask "what matter is it whether a man be born at Inverness or Dover; is he not in either case a British subject? And even if he were not, is not science of every country, &c." We would ask, in turn, would it not be grating to the feelings of Englishmen to be ruled by a knot of Scotchmen, however wise, learned, and decently behaved they might be? Putting aside all temporary pique, would any one like to see the English college of physicians converted into another India-house, and governed by the Frasers, the Donaldsons, the Mackenzies, the Murgatroyds, and their cousins, to the fortieth degree of affinity? We are quite sure that there is not one Englishman in a thousand who, in his cooler moments, would think the thing bearable. This cosmopolitan liberality may be worshipped with the lips, but every generous heart is far from it. Should these humble pages meet the eye of any member of the legislature, we would inquire of him what would be thought of an attempt to introduce at one blow only one hundred Scotchmen into his club—the Athenæum peradventure, or the Travellers'. Would not the west-end be distracted with anti-canvassing?—would not all the turners in town be overwhelmed with orders for black balls, at the very sound of such a proposal?

In a word, then, the objections to the indiscriminate admission of M.D.'s, to the fellowship of the college, are chiefly two: first, a majority of the governing body would consist of Scotchmen; and, secondly, from the extreme facility with which many universities grant degrees, this *upper house*, which should represent the whole body of physicians in general society and intellectual warfare, would contain a large admixture of persons, who, though possibly not unskilled in the practical parts of the medical art, could hardly be called members of a learned profession. We shall return to this subject in our next number.

